# Effectiveness of a Program Based on Action-Observation Training (AOT) on Motor, Functional and Cognitive Aspects in Patients with Cognitive Impairment: A Non-Randomized Controlled Trial

**DOI:** 10.3390/healthcare11071030

**Published:** 2023-04-04

**Authors:** Cecilia Estrada-Barranco, Maria de los Ángeles Martinez-Javaloyes, Isabel Rodriguez-Costa, Ismael Sanz-Esteban, Alberto Bermejo-Franco, Araceli Aranda-Ruiperez, Maria de los Ángeles Gallegos-Martínez

**Affiliations:** 1 Department of Physiotherapy, Faculty of Sport Sciences, Universidad Europea de Madrid, Villaviciosa de Odón, 28670 Madrid, Spain; 2 Humanization in the Intervention of Physiotherapy for the Integral Attention to the People (HIPATIA) Research Group, Physiotherapy Department, Faculty of Medicine and Health Sciences, University of Alcalá, Alcalá de Henares, 28805 Madrid, Spain; 3 Residencia Hermandad Nuestra Señora de la Soledad, Parla, 28981 Madrid, Spain

**Keywords:** action-observation treatment, dementia, cognitive impairment, mirror neurons, elderly people

## Abstract

**Highlight:**

- The AOT facilitates motor learning in patients with cognitive impairment.
- Intervention with AOT in patients with cognitive impairment may be effective in improving functionality of their dominant upper limb and independence in basic activities of daily living.

**What is the implication of the main finding?**

- AOT could be an effective therapeutic tool in the approach to older adults with cognitive imparirment.
- Group therapy itself may be a determining factor in the therapeutic approach for patients with cognitive impairment.

**Abstract:**

Cognitive impairment is frequent in elderly subjects. It is associated with motor impairment, a limitation in quality of life and frequently, institutionalization. The aim of this work is to test the efficacy of a therapeutic group program based on action-observation learning. Methods: a non-randomized controlled trial study was conducted. We included 40 patients with cognitive impairment from a nursing home who were categorized into mild and moderate cognitive impairment and divided separately into a control and experimental group. Experimental group performed a 4-week group work, in which each patient with mild cognitive impairment was paired with a patient with moderate cognitive impairment. Thus, patients with mild cognitive impairment observed a series of functional exercises performed by their peers and replicated them. Simultaneously, the patients with moderate cognitive impairment replicated the movement after observing it performed by a patient with mild cognitive impairment. The control group continued to receive their usual care at the center. The upper limb function, cognitive level and function in basic activities of human daily life were measured before and after the intervention and compared with the control group. Results: statistically significant differences were found in the functionality of basic activities of daily living, in the functionality of the upper limb and in the cognitive level in all patients in the experimental group regardless of the initial cognitive level. No statistically significant differences were found in the control group. Conclusions: the implementation of a group, peer-based, action-observation learning therapeutic program is effective in improving the basic activities of human daily life, cognitive level and upper limb functionality in patients with mild and moderate dementia.

## 1. Introduction

Cognitive impairment is defined as a clinical entity in which intellectual functions are partially or totally modified leading to alterations in memory, attention and speed of information processing [1,2]. Physiological, biochemical, metabolic and morphological changes that occur during ageing can threaten this capacity in older adults. Cognitive impairment can be considered one of the major functional consequences of ageing in addition to other changes, such as, sensory and motor deficits, which threaten the functionality and thus the independence of older adults [1]. The term mild cognitive impairment (MCI), as defined by the American Psychiatric Association (APA 1987), is a condition that includes short-term and long-term memory impairment, but not functional impairment. MCI is a precursor to dementia [3].

According to the World Health Organization (WHO), in the population over 65, an estimated 5.0 million adults were affected by cognitive impairment in 2014 and this figure is projected to grow to nearly 14 million by 2060 [4]. The rate of patients with dementia increases significantly with increasing age, reaching 22.9% in patients over 85 years [4]. The progressive ageing of the population, with WHO predicting a doubling of the population over 60 by 2050, and the increase in life expectancy, will increase the number of patients with cognitive impairment in the coming years [5].

The performance of activities of daily living is achieved by a fusion of motor, cognitive, and socioemotional skills [6]. Therefore, cognitive impairment can directly decrease independence for the basic activities of daily living (BADL), promote immobility and social isolation [7,8,9]. All these factors mean that patients with cognitive impairment are more likely to require admission to specialized centers.

Different therapeutic approaches based on physical activity have been shown to be effective for improving cognitive impairment. Luo et al. observed an improvement in cognitive impairment through an exercise program [10]. Tai Chi has also proved to be effective in treating patients with cognitive impairment [11]. Physical activity in general has been shown to be effective for improving functionality and strength in elderly patients with cognitive impairment [12]. However, limitations for physical activity in these patients are evident due to the difficulty in understanding tasks or the risk of falls.

Action-observation therapy (AOT) consists of observing a motor gesture performed by another subject and then proceeding to perform the same movement. This technique has been developed as a physical rehabilitation approach that promotes the brain’s plasticity by activating the mirror neuron system [13]. AOT has been shown to be effective in improving functionality, brain damage-associated cognitive impairment and activities of daily living in stroke patients [14,15] and in other pathologies, such as, cerebral palsy [16], as well as in reducing symptoms in pathologies such as Parkinson’s disease [17]. It has been shown that activation of the mirror neuron system is altered in patients with cognitive impairment [18]. For this reason, AOT could be useful for activating the mirror neuron mechanism, which is essential for motor learning [19].

Furthermore, it has been demonstrated that AOT is more effective when subjects observe an activity performed by individuals with similar characteristics to themselves. Research suggests that patients with the same condition, but at a lower degree of severity, who observe their peers performing a task, learn better than when they observe a physical therapist or another person without motor impairment [16]. Moreover, the social interaction that occurs during peer learning contributes to increased motivation and better adherence to the treatment [20]. This is particularly significant for institutionalized patients with cognitive impairment, given that social isolation is a common feature of the condition [21]. Rojasvastera et al. [22] demonstrated the effectiveness of AOT in improving gait and cognitive impairment in patients with cognitive impairment. However, as far as we are aware, the efficacy of AOT on the upper limb function and independent basic activities of daily living (BADL) in patients with cognitive impairment has not yet been investigated.

The aim of this study is to quantify the benefits of a group program based on action observation and in peer learning on the upper limb motor control/function, BADL independence and cognitive function in patients with mild and moderate cognitive impairment.

## 2. Materials and Methods

A non-randomized controlled trial was conducted to assess the impact on upper limb functionality, independence in activities of daily living (ADL), and cognitive function following the implementation of a physiotherapeutic intervention based on action observation in patients with mild and moderate dementia compared to the control group. The intervention was carried out between April and March 2022, over a period of 4 weeks. This study was approved by the local ethics committee: 22/178-E_TFM and was registered in the clinical trials registry NCT05585424. 

### 2.1. Setting and Participants

The sample was selected from the users of the nursing home “Nuestra Señora de la Soledad” in Madrid. The following inclusion criteria were established: users of the nursing home; over 65; mild or moderate cognitive impairment, with a mini cognitive examination (MEC) score of >13 and ≤24; patients participating in the physical and cognitive therapeutic activities carried out at the nursing home; patients requiring supervision or assistance for ADL. The following exclusion criteria were applied: patients with a musculoskeletal pathology of the dominant upper limb; presentation of visual deficits not correctable with glasses; patients with aggressive behavioral alterations or emotional lability; presentation of severe hypoacusis not correctable with hearing aids; patients who have had a stroke or severe neurological disease; and patients with any orthopedic problems.

Patients were classified according to the MEC scale score. Based on previous studies and the authors’ own description of the scale, patients with an MEC score of ≥13 and ≤19 were considered moderately cognitively impaired (ModCI), and those with a score of ≥19 and ≤23 were considered mildly cognitively impaired (MCI) [22,23,24,25].

The intervention lasted for 4 weeks, with a frequency of 3 times per week. It consisted of an upper limb functional exercise program performed in a seated position, based on AOT. Patients in the experimental group were paired so that each patient with MCI was partnered with a patient with ModCI. During the sessions, two therapists performed the upper limb activities, which only the MCI patients could see, and they performed them by imitation. The ModCI patients imitated the movement they observed in their MCI partner without seeing the therapists. The exercises progressively increased in difficulty each week as follows: the first week involved global upper limb mobilization exercises; the second week involved exercises with muscle synergy work and midline crossing; the third week involved resistance exercises; and the fourth week involved precision, fine gripper, and coordination exercises.

All variables were collected before and after the 4-week intervention by an independent researcher who was not involved in the intervention. Upper limb functionality was assessed with the Fugl-Meyer (FM) scale [26], independence for BADL was assessed with the Barthel index (BI) [27] and the Spanish version of the Mini-Examen Cognoscitivo (MEC) [28] was used to assess cognitive function. The CG was assessed at baseline and after one month, without any additional intervention, returned to normal care. All participants, both in the CG and EG, continued with their usual activities in the nursing home.

The Mini-Examen Cognoscitivo (MEC) [28,29] is the Spanish adaptation of the Mini-Mental State Examination (MMSE) [30]. It examines different cognitive functions: orientation, memory, attention, calculation, language, construction, praxis and reasoning. A score of 23 or below is associated with cognitive impairment [31].

The Fugl-Meyer scale (FM) was developed in 1975 to assess the motor function in stroke patients [26]. It was later validated for a non-specific population [32]. It can be divided into sections: upper limb, lower limb, balance, sensation, passive range of motion and joint pain. The upper limb subscale assesses sensation; joint movement and pain, and can be assessed without the need for a full assessment. The upper limb subsection has 33 items, with the maximum score of 66 being associated with the best upper limb motor function and 0 being the minimum score [26].

The Barthel Index (BI) assesses basic activities of daily living which includes tasks related to dressing and undressing, toileting, the ability to transfer to a chair or bed, urinary and fecal continence, the ability to use the toilet, ambulation and the ability to go up and down stairs. In total there are 10 items that add up to 5 or 10 points and total up to a maximum of 100 when the person is independent in terms of BADL [33,34]. It has proven its validity and reliability for assessing the functionality of elderly patients [35].

### 2.2. Sample Size

The construct validity sample size was calculated using the G*Power software (version G*Power 3.1.9.2). We established the following parameters to obtain the sample size using the Wilcoxon signed-rank test: one tailed, an error alfa of 0.05 [36] and a power of 0.95 [37], resulting in a sample size requirement of 47 participants.

### 2.3. Data Analysis

SPSS v.22 was used for the statistical analysis. The normality of the sample was calculated using the Shapiro-Wilk test and it could not be established that data followed a normal distribution. To analyze the differences between the control and experimental groups, the U-Mann Whitney test for independent samples was used. To compare the initial and final scores of the variables studied, the Wilcoxon signed-rank test was used.

## 3. Results

**Figure 1:**
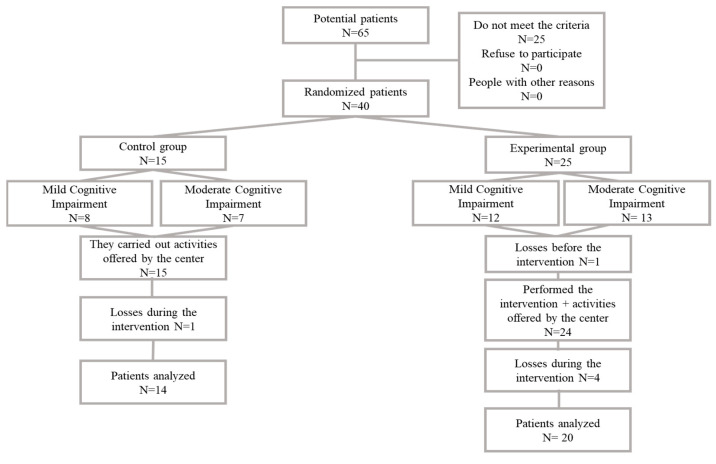
Flow diagram.

Sixty-five nursing home users were selected, of whom 40 met the inclusion criteria and were included in the study. Finally, 34 patients completed the study. Four losses were recorded: one for transfer to another center, two for illness and one for death. Patients were classified according to their cognitive impairment into patients with mild cognitive impairment (MCI) and patients with moderate cognitive impairment (ModCI) and assigned to the control group or the experimental group (Figure 1).

The general characteristics of the sample are presented in Table 1.

No statistically significant differences were found between the initial scores of the variables studied: MEC and BI of the CG with respect to EG. Nor was there any difference with respect to age in both groups. Differences were found in the initial FM score in the group of subjects with moderate cognitive impairment between the control and experimental groups. The FM score in the MDG group was not collected. It was calculated using the Mann-Whitney U test, establishing a statistical significance value of *p* < 0.05 (Table 2).

After 4 weeks, results were collected for all three variables. Improvements in BADL independence, cognitive status and upper limb function were compared using the difference in scores at baseline and at the end of the intervention. Statistically significant differences were found in the results of independence for BADL (BI), cognitive status (MEC) and upper limb function (FM) between pre-intervention and post-intervention measurements in the EG. The Wilcoxon test for paired samples was used, but no differences were found in the CG with a statistical significance value of *p* < 0.05 (Table 3).

Furthermore, no statistically significant differences were found between the improvement found in patients with MCI and patients with modCI in the EG in the independence for BADL (BI) and cognitive status (MEC) tests. Upper limb functionality (FM) was not collected in patients with mild cognitive impairment. These differences were compared using the Mann Whitney U-test for independent samples. A value of *p* < 0.05 was established as statistically significant (Table 4).

## 4. Discussion

According to our results, all patients in the EG, in contrast to patients in the CG, improved their functional independence and their cognitive function. In addition, patients with moderate cognitive impairment improved their upper limb functionality. Patients with modCI did not improve more than patients with MCI.

The results obtained could be explained by the findings of the experimental studies that show that when observing an action, the same brain areas, the dorsal and ventral premotor areas and the presupplementary motor area, are recruited as when the action is performed [38,39]; and, furthermore, activation in areas involved in motor preparation and execution is stronger when the action is observed prior to its performance [40], thus enhancing motor learning through the mirror neuron system.

Since many patients are wheelchair users, we chose to work with activities involving the upper limbs. However, improved motor control in this segment is directly related to independence in performing BADL [41,42]. AOT is based on the mirror neuron mechanism and recent evidence shows that this neural activation mechanism is not only involved in simple activities but is also activated in more complex tasks such as manual functions [43].

The exercises performed were shown in real time and through movement by two therapists. AOT has been shown to be more effective when performed through dynamic instructions than teaching by static means such as pictures [44]. In addition, exercises varied during sessions and progressed through the weeks, as there is a stronger activation of the mirror neuron system in new activities than in previously practiced ones [45]. Pair formation was randomized to ensure that their previous relationship did not affect the results of the study and may differ from other research, such as, Naura et al. [16] who worked with patients from home and were matched according to their age to improve their bonding [16]. Other works carried out through AOT also used recorded images to observe the action. However, in our study, patients interacted with each other during the sessions, which generated a connection that could favor treatment adherence and outcomes [16].

Physical activity, of various types, has been demonstrated to be effective in enhancing functionality, cognitive impairment and motor function. However, determining the most effective type of activity or therapy has been challenging [10,11,12]. The application of physical therapy in patients with cognitive impairment is limited. AOT has a key characteristic that sets it apart: it does not require patients to comprehend verbal instructions or understand the exercises or activities to be performed explicitly. The subject only has to repeat what they see. This aspect can be critical in patients with cognitive impairment, as comprehension may be impaired.

The benefits of this intervention have been shown to have positive benefits in both groups, with moderate and mild cognitive impairment. This may be due to the fact that work was carried out in a group. Group treatments have other benefits in terms of motivation and open up an interesting possibility for intervention in nursing homes [46]. The fact that significant changes have been obtained in comparison with users in their usual practice makes it possible to consider a specific intervention without an increase in human and material resources.

In addition to this association, pairing and group sessions are supported by previous research, such as that of Kuiper et al., in which they concluded that physical inactivity and poor social interaction were established risks for the onset and increase of dementia [47], which could justify the fact that, after treatment, patients improved their cognitive level.

During the sessions, patients with modCI observed their partner with MCI who, in turn, observed and performed the movement made by the therapist. The previous studies support the fact that, learning is greater when observing a subject with similar motor skills and the action is feasible for the subject, albeit, with errors [48]. Rohbanfard et al. also corroborated that the maximum improvement is attained through AOT when a movement with a certain range of error is observed [49]. According to our results, there was no difference in improvement between the groups with mild and moderate cognitive impairment. This can be explained since the group of patients with MCI copied the movement performed by the therapists and the patients with modCI were able to learn from observing the patients with mild cognitive impairment.

The intervention lasted four weeks. Other studies, such as Ertelt et al., also showed significant changes after four weeks of intervention with AOT and even showed that they were maintained for at least 8 weeks after the end of treatment [50]. However, Castro et al. in their systematic review discuss the effectiveness of physical activity in decreasing cognitive impairment by comparing studies with interventions lasting over 3 months [51].

This study has limitations. As there were more losses than expected, the sample size was not sufficiently large to allow extrapolating the results. Studies with a larger sample size are needed. Therefore, in the group with ModCI, learning from direct observation by the therapist was not compared, and it cannot be concluded from our results that this intervention was more effective than the other intervention. This work did not involve any activities that included the lower limb or balance. Since the FM data were not collected for patients with mild cognitive impairment, a comparison of improvement between subjects with MCI versus modCI could not be made. Future research should take this into account. Future research should also consider these aspects to evaluate the effect of a program with these characteristics on balance, gait or community participation.

In addition, the long-term effects of the program on the patient as well as the results of a longer intervention could be lines of future research. Future studies should study the gender difference in AOT intervention.

## 5. Conclusions

Intervention with AOT based on the stimulation of mirror neurons was beneficial for patients with cognitive impairment, increasing the functionality of their dominant upper limb, cognition and independence in BADL. However, future research should confirm these findings by expanding the sample size, extending the intervention period, and assessing the long-term sustainability of these benefits.

## Figures and Tables

**Table 1:** Sample characteristics.

	CG		EG	
	Mild CI	Moderate CI	Mild CI	Moderate CI
N	8	7	12	13
Age, y (m (DS))	87.38 (3.48)	82.43 (5.25)	89.08 (5.63)	86.92 (7.65)
Sex, n	6 F; 2 M	4 F; 3 M	11 F; 1 M	10 F; 3 M

CG: Control group; EG: Experimental group; CI: cognitive impairment; y: years; m: media; SD: standard deviation.

**Table 2:** Initial comparison between CG and EG.

MEC Initial		BI Initial		FM Initial modCI		Edad	
Median (IR)	*p*	Median (IR)	*p*	Median (IR)	*p*	Median (IR)	*p*
18 (5)	0.265	45 (28)	0.300	99 (36)	0.02	87 (8)	0.165

MEC: Mini-Examen Cognoscitivo; BI: Barthel index; FM: Fugl-Meyer; modCI: moderate cognitive impairment; IR: interquartile range; *p*: *p* value.

**Table 3:** Comparison of initial and final CG and EG.

	CG	EG
	*p*	*p*
IB	0.157	0.001
MEC	0.461	0.001
FM	0.925	0.008

CG: Control group; EG: experimental group; IB: Barthel index; MEC: Mini-Examen Cognoscitivo; FM: Fugl-Meyer; *p*: *p* value.

**Table 4:** Comparison of the improvement of patients with mild and moderate dementia in EG.

IB			MEC		
MCI	ModCI	*p*	MCI	ModCI	*p*
Median (IR)	Median (IR)		Median (IR)	Median (IR)	
5 (5)	5 (5)	0.632	1.5 (2.25)	1 (3)	0.745

MCI: mild cognitive impairment; moderate cognitive impairment; BI: Barthel index; MEC: Mini-Examen Cognoscitivo; IR: interquartile range; *p*: *p* value.

## Data Availability

All data is presented in the study. If you need any additional information, you can write corresponding author (cecilia.estrada@universidadeuropea.es).
